# Family Fractures: A Multilevel Analysis of Institutional Drivers Behind Elderly Empty-Nesting in China’s Rust Belt

**DOI:** 10.1093/geroni/igaf122.2828

**Published:** 2025-12-31

**Authors:** Xiaoxuan Liang, Gong Chen, Bo Liang

**Affiliations:** Peking University, Beijing, Beijing, China (People’s Republic); Peking University, Beijing, Beijing, China (People’s Republic); Peking University, Beijing, Beijing, China (People’s Republic)

## Abstract

This study employs a multilevel institutional analysis to investigate the structural disintegration of intergenerational support systems and the rising prevalence of elderly empty-nesting in China’s Rust Belt (Northeast provinces), utilizing longitudinal population census data (2000–2020) and municipal socioeconomic records. Three critical findings emerge: (1) Accelerated Family Fractures: The Rust Belt exhibits a uniquely severe trajectory of aging and empty-nesting compared to national averages. While couple-only households dominate elderly empty-nesting (reflecting weakened intergenerational cohabitation norms), the surge in solitary living arrangements—a 23% faster growth rate than couple-only cases—signals deepening familial fragmentation. (2) Spatial Inequality as Institutional Legacy: Urban empty-nesting rates persistently surpass rural levels, yet resource-depleted cities face disproportionately high rates of solitary elderly households. Geospatial clustering reveals a “peripheralization” pattern: border zones of Heilongjiang-Jilin and industrial hubs in Liaoning exhibit peak empty-nesting, mirroring the region’s post-industrial decline and youth exodus. (3) Hierarchical Drivers of Institutional Failure: Mass outmigration (demographic driver) and state-led urbanization policies (economic driver) structurally erode family support capacities. The collapse of multigenerational cohabitation norms, amplified by decades of one-child policy legacies, accelerates household atomization. Solitary living correlates strongly with localized industrial collapse (e.g., mining towns), whereas couple-only households are primarily shaped by cultural inertia and demographic constraints. These findings challenge conventional narratives of filial piety decline, instead framing empty-nesting as a systemic outcome of China’s Rust Belt’s institutional trilemma: depopulation pressures, fragmented welfare systems, and unaddressed cultural-institutional voids.

